# Semantics, Specification, and Bounded Verification of Concurrent Libraries in Replicated Systems

**DOI:** 10.1007/978-3-030-53288-8_13

**Published:** 2020-06-13

**Authors:** Kartik Nagar, Prasita Mukherjee, Suresh Jagannathan

**Affiliations:** 8 Microsoft Research Lab, Redmond, WA USA; 9 University of Southern California, Los Angeles, CA USA; 10 IIT Madras, Chennai, India; 11 Purdue University, West Lafayette, USA

## Abstract

Geo-replicated systems provide a number of desirable properties such as globally low latency, high availability, scalability, and built-in fault tolerance. Unfortunately, programming correct applications on top of such systems has proven to be very challenging, in large part because of the weak consistency guarantees they offer. These complexities are exacerbated when we try to adapt existing highly-performant concurrent libraries developed for shared-memory environments to this setting. The use of these libraries, developed with performance and scalability in mind, is highly desirable. But, identifying a suitable notion of correctness to check their validity under a weakly consistent execution model has not been well-studied, in large part because it is problematic to naïvely transplant criteria such as linearizability that has a useful interpretation in a shared-memory context to a distributed one where the cost of imposing a (logical) global ordering on all actions is prohibitive. In this paper, we tackle these issues by proposing appropriate semantics and specifications for highly-concurrent libraries in a weakly-consistent, replicated setting. We use these specifications to develop a static analysis framework that can automatically detect correctness violations of library implementations parameterized with respect to the different consistency policies provided by the underlying system. We use our framework to analyze the behavior of a number of highly non-trivial library implementations of stacks, queues, and exchangers. Our results provide the first demonstration that automated correctness checking of concurrent libraries in a weakly geo-replicated setting is both feasible and practical.

## Introduction

Geo-replicated systems maintain multiple copies of data at different locations and provide a number of attractive properties such as globally uniform low access-latency, always-on availability, fault tolerance, and improved scalability. Applications with a geo-distributed user base need to necessarily run on top of replicated systems to ensure fast and always-available service. On the other hand, due to concurrent updates at different replicas and the possibility of arbitrary re-ordering of updates by the underlying network, replicated systems typically guarantee a very weak form of consistency called *eventual consistency* [4], that only requires replicas which have received the same set of updates to exhibit the same state. Because this guarantee is often too weak to satisfy an application’s correctness requirements, a number of (stronger) consistency policies have emerged in recent years; these policies offer session [39], causality [27] or transactional [13] guarantees, and constrain system behavior by imposing additional synchronization on actions. Nonetheless, writing correct applications in this environment using these policies remains a challenging problem.

Having a library of performant *and correct* data structure implementations developed with replication and geo-distribution in mind can significantly alleviate the problem of writing correct applications, as demonstrated by the availability of highly popular concurrent library implementations developed for shared-memory systems [21, 33]. CRDTs [36] (Conflict-Free Replicated Data Types) offer an analog of such implementations for geo-replicated environments. However, using CRDTS to build useful data structure libraries is challenging because the strong requirements imposed by CRDTs (namely that all operations commute with each other) appears satisfiable only for simple objects such as sets, lists, or maps. Important data structures such as stacks, queues, or exchangers that serve as building blocks for many concurrent and distributed algorithms have eluded implementations using CRDTs. Even when a data structure can be expressed in this way, reasoning about its correctness is typically given in terms of non-standard criteria such as replicated data type specifications [12], convergence [31] or replication-aware linearizability [41], concepts that are likely to be difficult for programmers to grasp, especially when contrasted with well-established notions such as linearizability used to reason about shared-memory concurrency. This state of affairs has made it difficult to seamlessly adapt and exploit ongoing progress in the development of scalable and correct concurrent algorithms used in the shared-memory world to a geo-replicated setting.

In order to bridge this gap, we study how to *automatically transplant* concurrent library implementations developed for shared memory systems to replicated ones. Doing so would allow us to use carefully-crafted implementations which have been proven to run correctly in shared memory environments, thereby simplifying the task of building distributed replication-aware applications. However, realizing this goal poses a number of challenges, the most critical of which is the widely different memory consistency models used in the two domains: the eventually consistent memory model typically provided by a replicated system is significantly weaker than the sequential consistency guarantees offered by shared-memory. Consistency policies offering session, causal, or transactional guarantees must be additionally considered to facilitate correct behavior. This requires enriching the *semantics* of existing library implementations to take into account the consistency policy of the underlying replicated system. Furthermore, the *de facto* correctness criterion for concurrent library implementations is linearizability, which is clearly too restrictive to be directly applied to this much weaker setting, since it demands that any correct execution be equivalent to some sequential execution of a reference implementation. Such a requirement is problematic in a geo-replicated environment where the cost of coordination to enforce a global ordering of all actions is prohibitive. These observations are similar to those made by Raad et al. [34] who considered the applicability of linearizability in a weak memory context, a scenario that faces similar challenges to our own. To address these issues, we therefore consider alternative declarative specifications of data structures, based on axiomatic definitions [17], that are roughly equivalent to the guarantees provided by linearizability (and hence familiar to programmers), but suitably relaxed to take into account the weak behaviors admitted by replicated systems.

We then propose an automated approach to find bounded violations of these declarative specifications given an implementation and a consistency policy. Due to the non-deterministic nature of replicated systems, manifesting violations in actual executions requires (1) a specific combination of library methods to be called (2) with specific argument values and (3) a specific interaction of low-level read/write events. Indeed, existing approaches to checking application safety under weak consistency [24] potentially involve long (on the order of hours) and costly execution runs to offer meaningful assurance on application correctness given the large space of possible behaviors that can be exhibited.

In contrast to testing approaches, our analysis framework directly searches for an execution violating a specification, and in the process *constructs* the combination of library methods to be called as well as their argument values, and the low-level read/writes which can lead to the violation. Moreover, because our analysis is parametric in the choice of consistency policy, we can constrain the search for violating executions on-demand as per the chosen policy. We additionally show how our technique is capable of expressing complex correctness specifications of libraries (see Sect. 3.4) and how it can be used to automatically find violations in the face of this complexity. The analysis is sound in that it only reports actual violations. Notably, our experiments manifest a number of non-trivial and complex violating executions for realistic concurrent libraries which require intricate interaction with library methods. We were also able to analyse application behavior under different consistency policies, and in particular, were able to find the weakest consistency policy to eliminate a particular violation. Our analysis is based on developing an efficient encoding of the implementation, the consistency policy, and the correctness specification as first-order logic formulae which can be dispatched to off-the-shelf SMT solvers to find violations. Unlike random testing approaches, our technique is capable of identifying non-trivial subtle safety violations in the order of minutes, making it feasible to use not only for finding violations, but also for checking the feasibility of any proposed remediations. We make the following major contributions:

1. We propose a novel operational semantics for replicated systems parameterized under realistic consistency policies which can be used to describe executions of sophisticated concurrent library implementations.
2. We demonstrate how to adapt existing specification frameworks developed for concurrent libraries on shared memory systems to replicated systems with minimal changes.
3. We describe an automated bounded verification procedure to detect violations of such specifications for implementations intended to execute under a given consistency policy.
4. We catalog the results of applying our analysis on a number of well-studied implementations including stacks, queues and exchangers, on a commercial replicated store (Cassandra), demonstrating empirically that our correctness checking procedure is useful in practice.

The remainder of the paper is organized as follows. In the next section, we provide a motivating example to illustrate the challenges of reasoning about concurrent libraries in a weakly-consistent replicated environment. Section 3 formalizes the language used to write library implementations and the specifications that characterize their intended behavior. Section 4 describes our bounded verification procedure and provides details about how we encode extracted verification conditions. Section 5 describes experimental results and presents case studies to illustrate the effectiveness of our approach. Related work and conclusions are given in Sect. 6.

## Illustrative Example

**Fig. 1.**
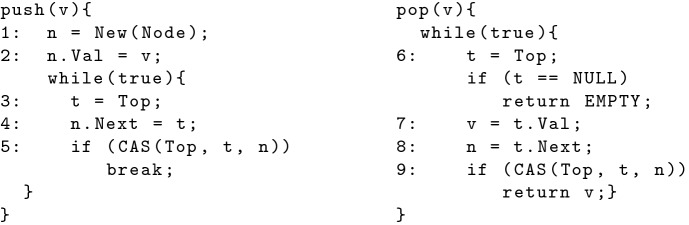
Treiber Stack

In this section, we illustrate the various issues that arise when running standard concurrent library implementations on replicated systems. Figure 1 shows the implementation of a Treiber stack, suitably adapted to execute in a replicated environment. The Treiber stack provides two methods (push and pop) to clients, and stores the elements of the stack in a linked list, with the order of elements in the list corresponding to the order in which elements are pushed. Since replicated stores typically offer a database or a key-value store interface, we store the linked list as a table of type Node with columns Val and Next, where each row stores a node of the linked list, with Val storing the value and Next storing the id of the next node. Top contains the id of the Node row which is current top of the stack (Top is initialized with the special value NULL indicating an empty stack). In Fig. 1, variables denoted by lower-case letters are assumed to be stored locally and are not replicated. New(Node) returns the id of a new row in the Node table. CAS(Top, t, n) is the typical Compare-And-Swap operation which atomically compares Top to t, and if it is equal to t then updates it to n^1^.

Clients of concurrent libraries issue invocations of a data structure’s methods, possibly at different replicas, with invocations being grouped together into *sessions*, with each session containing invocations issued by the same client. Whenever a method is invoked, the underlying implementation of the method is executed; we assume the various reads and writes performed by the method may possibly be executed at different replicas. All low-level operations performed by the same invocation are defined to be in the same session (i.e. the session of the parent invocation). Notice that the implementation stores data across a number of locations (e.g. Top or a cell in the Node table), each of which are operated independently through low-level read/write/CAS operations. The replicated store only guarantees eventual consistency, which means that the values stored at all locations eventually converge across all replicas. However, users expect the behavior of the library to conform to the specification of the stack data structure, regardless of when and how updates propagate across replicas.

**Fig. 2.**
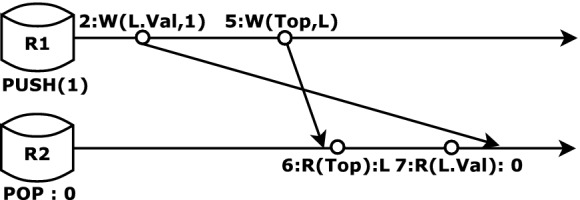
An execution of Treiber Stack on a replicated store

Consider the following basic specification (adapted from the $\mathsf {AddRem}$ axiom in [17]), which simply says that any value returned by a $\mathsf {POP}$ operation must have been pushed by some $\mathsf {PUSH}$ operation in the execution; observe that the specification does not allude to any specific system-level issues related to replication or weak consistency:

$$ \forall \gamma . \mathsf {meth}(\gamma ) = \texttt {POP}\ \wedge \mathsf {ret}(\gamma ) \ne \texttt {EMPTY}\ \Rightarrow \exists \gamma '. \mathsf {meth}(\gamma ') = \texttt {PUSH}\ \wedge \mathsf {arg}(\gamma ') = \mathsf {ret}(\gamma ) $$

Consider the execution shown in Fig. 2 that involves an invocation of $\mathsf {PUSH}(1)$ and $\mathsf {POP}$ from two different replicas. Among the many operations that the implementation of $\mathsf {PUSH}$ performs, we show only two write operations in the figure (along with line numbers referring to the implementation in Fig. 1), namely the write to the Val field of location $\mathsf {L}$ ($\mathsf {L}$ is the id of the new Node), and the write to Top as a result of the successful CAS. Similarly, for the $\mathsf {POP}$ operation, we show the read to Top, and then the read to the Val field. In the execution, the write to Top propagates from replica R1 to R2 before the read, but the write to Val does not, so that $\mathsf {POP}$ sees that a new node has been pushed but does not read the value that was actually pushed, instead returning the initial value of the location, thus breaking the specification described above. Eventual consistency only guarantees that eventually, the write to Val will also be propagated to R2, which is not sufficient to guarantee the specification holds under all executions.

One way to avoid this counterexample would be to ensure that the write to Val field by $\mathsf {PUSH}$ is propagated to another replica before the write to Top, thus guaranteeing that it would be available to the read of Val by $\mathsf {POP}$. Notice that the write to Val occurs before the write to Top in the same session, and hence we can use session guarantees to ensure the required behavior. In particular, under a *Monotonic Writes* (MW) consistency policy, writes are always propagated in their session order to all replicas [1]. However, MW is not sufficient by itself to eliminate the counterexample since the reads to Top and Val by $\mathsf {POP}$ may occur at different replicas, so that the read to Val may occur at a replica in which none of the writes by $\mathsf {PUSH}$ have propagated. Hence, we also need to have these operations execute under a *Monotonic Reads* (MR) consistency policy that mandates all writes witnessed by an operation will also be witnessed by later operations in the same session.^2^

**Fig. 3.**
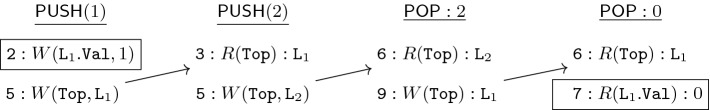
A violation of $\mathsf {AddRem}$ by Treiber Stack under MW+MR

Hence, a combination of MW+MR prevents the counterexample in Fig. 2, but it is unfortunately not enough to guarantee the $\mathsf {AddRem}$ specification is correctly enforced. Consider the execution in Fig. 3 which involves four method invocations (2 $\texttt {Push}$es and 2 Pops), where each invocation occurs on a different replica. Again, we only show some relevant low-level operations performed by these invocations, with arrows from write to read operations showing reads-from ($\mathsf {rf}$) dependencies. In the execution, after the two pushes, 2 is stored on the top of stack at Node $\texttt {L}_2$. Thus, the first Pop operation returns 2 and sets the Top to point at $\texttt {L}_1$, which is then read by the second Pop. However, MW+MR only guarantees that all write operations performed by the first Pop will be witnessed by the second Pop. Hence, just like in Fig. 2, the second Pop operation may see the node at location $\texttt {L}_1$ but not the write to the Val field (which was performed by PUSH(1)), resulting in violation of the specification. To avoid this, it must be guaranteed that the write to $\texttt {L}_1.\texttt {Val}$ by Push(1) must be visible to its read by the second Pop (depicted by the two boxes in Fig. 3). This can be guaranteed by the *Write Follows Read* (WFR) policy, which analogously to MW, ensures that writes witnessed in a session are propagated to all replicas before writes of the session itself (as opposed to MW which only ensures that writes performed in a session are propagated in session order). We note that both the violations described above (along with their repairs) were automatically discovered using our proposed methods, which devised solutions significantly less expensive than imposing strong consistency (aka global coordination) on all accesses.

While MW+MR+WFR is required to ensure $\mathsf {AddRem}$ in a Treiber Stack, we found that weaker consistency policies (including *Eventual Consistency*) were sufficient for other properties and benchmarks (more details are provided in Sect. 5).

## Semantics and Specifications

In this section, we define a simple language to write library implementations that is nonetheless powerful enough to express a number of real-world implementations. We then define an operational semantics to express executions of any implementation written in this language on top of a replicated store. A key feature of this operational semantics is that it is parametric in the consistency policies available to the store. Thus, instantiating the semantics with different consistency policy definitions allows us to reason about library behavior under replicated stores providing different consistency guarantees. Another important feature of the semantics is that it abstracts out low-level operational details such as the number of replicas, the specific manifestation of how message sends and receives are implemented, etc., and instead uses a succinct representation involving read and write events (and various binary relations among them) to capture salient characteristics sufficient to reason about library correctness with respect to consistency properties. The proposed semantics facilitates a bounded verification approach that is parametric in the consistency policy, and also matches very well with existing axiomatic approaches to specify correctness of library implementations in shared memory systems.

First, we define a simple imperative language in which implementations can be written:The only difference between standard shared-memory programs and those written in the above language is that read and write operations can now be performed on either Locations, which are replicated, or local variables which are not. As we saw in Sect. 2, replicated Locations can in general refer to any field in any table. Let $\mathbb {P}$ be the set of programs (*c*) generated using the above grammar. A **library** $L = (M, I)$ consists of a set of methods (*M*) and an implementation function $I : M \rightarrow \mathbb {P}$. For simplicity, we assume that each method takes as input one argument. Assume that *I*(*m*) contains the free variable $\texttt {a}$ that stores the input argument. Let $\mathbb {V}$ be the value domain for arguments and return values. We designate a special value $\bot \in \mathbb {V}$ for the cases where the argument or return value is empty.

The methods of a library implementation *L* can be invoked any number of times by multiple clients. Invocations from the same client are grouped together into **sessions**, where each session consists of a sequence of method invocations. Following standard terminology, given a set of sessions *S*, an interaction between clients and the library is expressed as a **history**, $h: S \rightarrow (M \times \mathbb {V})^*$, which simply associates a sequence of methods invocations to each session. An execution of the history corresponds to executing the library implementation of each method in the history on the replicated store. The store constrains the behavior of reads, writes and CAS operations to replicated Locations through its consistency policy.

We now formally define the operational semantics of a history on a replicated store that is parametric in a consistency policy $\varPsi$. While the history only associates arguments with method invocations, executing it on the replicated store will give rise to an **abstract execution**, which will also associate return values with invocations, and whose correctness we are interested in checking. Given a history *h*, library *L*, and consistency policy $\varPsi$, we define our semantics in terms of a labeled transition system (LTS) $\varOmega _{h,L,\varPsi } = (\varPhi , \mathcal {E}, \rightarrow )$, where $\varPhi$ denotes a set of states, $\mathcal {E}$ denotes a set of events (also used as labels) and $\rightarrow \subseteq \varPhi \times \mathcal {E} \times \varPhi$ defines a transition relation over states and events.

Each state in $\varPhi$ is specified as a tuple $(\chi , h', \mu , \texttt {c}, \alpha )$. $\chi$ denotes the replicated store state and consists of read/write/update events to Locations and various relations among them (described in detail later); $h' : S \rightarrow (M \times \mathbb {V})^*$ denotes the continuation of the history, i.e., the remaining history yet to be executed; $\mu : S \rightarrow (\texttt {LocalVar} \rightarrow \mathbb {V})$ denotes the local variables map for each session; $\texttt {c} : S \rightarrow \mathbb {P}$ denotes the continuation of the current invocation for each session, i.e., the implementation of the current invocation for each session that is yet to be executed and $\alpha$ denotes the abstract execution. Each **event** $\sigma \in \mathcal {E}$ is a tuple (*i*, *s*, *a*), where *i* is a unique event-id, $s \in S$ is the session from which the event originated, and *a* is the action to the replicated store (either read $\mathsf {R}(l,n)$, write $\mathsf {W}(l,n)$ or update $\mathsf {U}(l,m,n)$). Given an event $\sigma =(i,s,a)$, $act(\sigma )$ denotes the action *a*, $loc(\sigma )$ denotes the location that is the subject of the action.

### Language Semantics

To simplify the presentation, we decouple the semantics of the language from the semantics of the replicated store. The language is defined via a standard imperative semantics *except* that there are no constraints on reads to replicated locations (i.e., we do not mandate a specific replica that is targeted by the read), and every operation to a replicated location generates an event. These rules do not concern the replicated store state, and hence are of the form $(h_1, \mu _1, c_1, \alpha _1) \xrightarrow {\sigma } (h_2, \mu _2, c_2, \alpha _2)$ (i.e. omitting $\chi$ from $\varPhi$). We essentially pick any session and then execute the next operation from the current invocation in the session, or initiate the next invocation in the session if there is no invocation currently running. As an illustration, consider the following rule L-Read:

$$ \frac{\begin{array}{c}\texttt {c}(s) \equiv \texttt {v}=\texttt {l};\texttt {c}' \quad \sigma = (\mathsf {i},s,\mathsf {R}(l,n)) \quad \mathsf {fresh}\ \mathsf {i} \end{array}}{\begin{array}{c}(h',\mu ,\texttt {c},\alpha ) \xrightarrow {\sigma } (h', \mu [s \rightarrow \mu (s)[\texttt {v} \rightarrow n]], \texttt {c}[s \rightarrow \texttt {c}'],\alpha )\end{array}} $$

The rule picks the next operation in session $\mathsf {s}$ which is a read operation to location $\texttt {l}$, and generates the read event $\sigma$ reading value $\mathsf {n}$ from $\texttt {l}$. It updates the local variable $\texttt {v}$ to this value, leaving the yet-to-be-executed history ($h'$) and abstract execution ($\alpha$) unchanged. Write statements (i.e. $\texttt {l} = n$) generate write events ($\mathsf {W}(l,n)$), successful CAS statements (i.e. $\texttt {v} = \texttt {CAS}(\texttt {l}, m, n$) generate update events ($\mathsf {U}(l,m,n)$), and unsuccessful CAS generates read events ($\mathsf {R}(l,m')$). The complete set of rules can be found in the technical report [32].

### Abstract Execution Semantics

An **abstract execution** $\alpha = (\varGamma , \mathsf {so}_{\varGamma })$ maintains a set of method invocation events in $\varGamma$ and a session order relation $\mathsf {so}_{\varGamma }$ among these events. Each method invocation event $\gamma \in \varGamma$ is a tuple (*i*, *m*, *a*, *r*, *s*) where *i* is a unique event-id, $m \in M$ is a method of the library, $a,r \in \mathbb {V}$ are the method argument and return values respectively and $s \in S$ is the session from which the method was called. We use the notation $\varGamma ^{s}$ for the subset of $\varGamma$ which only contains method invocation events that originate in session *s*. The following rule (L-Return-Val) describes the generation of a method invocation event, which occurs on encountering a return statement during execution, and which is added to the abstract execution.

$$\frac{\begin{array}{c}\texttt {c}(s) \equiv \texttt {return } e;c' \quad h'(s) = m(k) \cdot h'' \quad \llbracket e \rrbracket _{\mu (s)} = n \\ \alpha = (\varGamma , \mathsf {so}_{\varGamma }) \quad \gamma = (i,m,k,n,s) \quad \alpha ' = (\varGamma \cup \{\gamma \}, \mathsf {so}_{\varGamma } \cup \varGamma ^{s} \times \{\gamma \} )\end{array}}{\begin{array}{c}(h', \mu , \texttt {c},\alpha ) \rightarrow (h'[s \rightarrow h''], \mu , \texttt {c}[s \rightarrow \epsilon ],\alpha ')\end{array}}$$

The rule updates the yet-to-be executed history $h'$ by removing the current invocation *m*(*k*) (since this invocation has now completed), updates the abstract execution $\alpha$ to now include the newly completed invocation, and updates the current invocation implementation to empty. Note that $\llbracket e \rrbracket \!_{\mu (s)}$ denotes the evaluation of the expression *e* under the local variable map $\mu (s)$. When the history $h'$ becomes empty, i.e. there are no more method invocations to be executed, the abstract execution becomes complete and would include all method instances present in the original history *h*. Note that this rule does not generate any read/write/update event.

### Replicated Store Semantics

The replicated store state $\chi = (\varSigma , \mathsf {vis}, \mathsf {ar}, \mathsf {so})$ consists of the set of replicated store events ($\varSigma$) and various relations on $\varSigma$. Events can either be read, write or update events, and depending on the type of event, $\varSigma$ is partitioned into $\varSigma _R, \varSigma _W$ and $\varSigma _U$. The visibility relation $\mathsf {vis} \subseteq \varSigma \times \varSigma$ denotes the events visible to an event and is used to determine the output of read events. The arbitration relation $\mathsf {ar} \subseteq (\varSigma _W \cup \varSigma _U) \times (\varSigma _W \cup \varSigma _U)$ provides a total ordering on write or update events to the same location. Finally, the session order relation $\mathsf {so} \subseteq \varSigma \times \varSigma$ provides a total ordering on events originating from the same session. All events generated by statements in the same method invocation would belong to the same session and hence would be related by $\mathsf {so}$. We also define a happens-before relation $\mathsf {hb} = (\mathsf {vis} \,\cup \, \mathsf {so})^{+}$ in the usual way.

We use $\varPsi$ to refer to a consistency policy supported by the store. $\varPsi$ is a predicate on the store state, which must be maintained at every step of the execution. $\varPsi$ essentially controls the visibility relation on events based on session or happens-before order. The following table illustrates the various consistency policies that we consider in our work; all of these policies can be implementation without the need for global coordination [1].^3^ (all $\sigma _i$ belong to $\varSigma$):

**Table 1.** Axiomatic characterization of various weak consistency policies.

Consistency policy	${\varPsi (\varSigma , \mathsf {vis}, \mathsf {ar}, \mathsf {so})}$
Read Your Writes [39]	$\mathsf {so}(\sigma _1, \sigma _2) \Rightarrow \mathsf {vis}(\sigma _1, \sigma _2)$
Monotonic Writes [39]	$\mathsf {so}(\sigma _1, \sigma _2) \wedge \mathsf {vis}(\sigma _2, \sigma _3) \Rightarrow \mathsf {vis}(\sigma _1, \sigma _3)$
Monotonic Reads [39]	$\mathsf {vis}(\sigma _1, \sigma _2) \wedge \mathsf {so}(\sigma _2, \sigma _3) \Rightarrow \mathsf {vis}(\sigma _1, \sigma _3)$
Write Follow Read [39]	$\mathsf {vis}(\sigma _1, \sigma _2) \wedge \mathsf {so}(\sigma _2, \sigma _3) \wedge \mathsf {vis}(\sigma _3, \sigma _4) \Rightarrow \mathsf {vis}(\sigma _1, \sigma _4)$
Causal Visibility [27]	$\mathsf {hb}(\sigma _1, \sigma _2) \wedge \mathsf {vis}(\sigma _2, \sigma _3) \Rightarrow \mathsf {vis}(\sigma _1, \sigma _3)$
Causal Consistency [27]	$\mathsf {hb}(\sigma _1, \sigma _2) \Rightarrow \mathsf {vis}(\sigma _1, \sigma _2)$

As we saw earlier in Sect. 2, $\mathsf{Monotonic Writes}$ enforces the constraint that if an event is visible, then all events before it in session order must also be visible. $\mathsf{Monotonic Reads}$ requires that if an event is visible, it will continue to remain visible to all operations later in the session. On the other hand, $\mathsf{Write Follows Read}$ enforces that all events visible to a prior event in a session will continue to remain visible to other events which witness a later event of the session.

We use the notation $\varSigma ^{l}$ to denote the subset of events pertaining to location *l*, and $\varSigma ^{s}$ to denote the subset of events of session *s*. Given a set of events $\varSigma '$, $\mathsf {MAX}^{\texttt {l}}_{\mathsf {ar}}(\varSigma ')$ denotes the maximal events in $\varSigma '$ according to the relation $\mathsf {ar}$ which write to location l. Given events $\sigma \in \varSigma _R^l$, $\sigma ' \in \varSigma _W^l$, we define the *Reads-From* relation $\mathsf {rf}$ in terms of $\mathsf {vis}$ and $\mathsf {ar}$ relations as follows:

$$\begin{aligned} \begin{array}{lcl} \mathsf {rf}(\sigma ',\sigma )\Leftrightarrow & {} \mathsf {vis}(\sigma ', \sigma ) \wedge \forall \sigma '' \in \varSigma ^l. (\mathsf {vis}(\sigma '',\sigma ) \wedge \sigma '' \ne \sigma ) \Rightarrow \mathsf {ar}(\sigma '', \sigma ')) \end{array} \end{aligned}$$

The $\mathsf {rf}$ relation essentially encodes the ‘last writer wins’ nature of the store, whereby the most recent visible write event according to $\mathsf {ar}$ becomes the event supplying the value available to subsequent reads. The replicated store state evolves by the addition of new events. On addition of a write/update event, the arbitration order is appropriately modified to ensure that it remains a total order on events targeting the same location. In addition, we also ensure causal arbitration [11] by enforcing that $\mathsf {ar}$ and $\mathsf {hb}$ do not disagree with each other. For update and read events, the values that these events read depend upon the most recent write event to the same location visible to the events, which in turn is controlled by the consistency policy. To elaborate, consider the rule R-CAS:

$${ \frac{\begin{array}{c}\varSigma ' \subseteq \varSigma \quad \sigma ' \in \mathsf {MAX}^{l}_{\mathsf {ar}}(\varSigma ') \quad \mathsf {ar} \subseteq \mathsf {ar}' \\ act(\sigma ') = \mathsf {W}(l,m) \vee act(\sigma ') = \mathsf {U}(l,\_,m) \quad \sigma = (i,s,U(l,m,n)) \quad \forall \tau \in \varSigma ^{l}_{U}. \lnot (\mathsf {rf}(\sigma ', \tau )) \\ \mathsf {ar}'\ \mathsf {is}\ \mathsf {a}\ \mathsf {total}\ \mathsf {order}\ \mathsf {on}\ \varSigma _W^l \cup \varSigma _U^l \cup \{\sigma \} \quad \forall \sigma _1,\sigma _2. \lnot (\mathsf {hb}(\sigma _1, \sigma _2) \wedge \mathsf {ar}'(\sigma _2, \sigma _1)) \\ vis' = vis \cup \varSigma ' \times \{\sigma \} \quad so' = so \cup \varSigma ^s \times \{\sigma \} \quad \varPsi (\varSigma \cup \{\sigma \}, \mathsf {vis}', \mathsf {ar}', \mathsf {so}')\end{array}}{\begin{array}{c} (\varSigma , \mathsf {vis}, \mathsf {ar}, \mathsf {so}) \xrightarrow {\sigma } (\varSigma \cup \{\sigma \},\mathsf {vis}',\mathsf {ar}',\mathsf {so}' )\end{array}}} $$

Here, we want to add a new update event to location *l*. First, an *arbitrary* subset ($\varSigma '$) of events of $\varSigma$ is selected. This step essentially corresponds to the creation of a new replica on which the events in $\varSigma '$ have been applied. Then, we select the most recent write event ($\sigma '$) from $\varSigma '$ which ensures atomicity of the update event (and hence the CAS statement responsible for the update). In particular, we require that no other update event must have read from ($\mathsf {rf}$) $\sigma '$. The value written by $\sigma '$ (i.e. *m*) would be the read value of the update event. $\mathsf {vis}$, $\mathsf {so}$ and $\mathsf {ar}$ are appropriately updated, and the new store state must satisfy the consistency policy $\varPsi$, which in turn will govern the selection of the initial subset $\varSigma '$. The formal rules for read and write events can be found in [32].

Note that enforcing the above rule would in essence prohibit two CAS operations to be executed concurrently, and hence would establish a global ordering among the CAS operations. However, unlike in shared memory systems where this is sufficient to establish a global ordering among all operations thus ensuring linearizability, in replicated systems, this does not constrain the behavior of other read and write operations (as we saw in Sect. 2, and hence more constraints must be enforced through the consistency policy.

We can now combine the language, abstract execution, and replicated store rules to describe transitions of the LTS $\varOmega _{h,L,\varPsi }$, which simply requires the language rules and the replicated store rules to agree on the structure of all replicated store events:

$$ \frac{\begin{array}{c}(h', \mu , \texttt {c}, \alpha ) \xrightarrow {\sigma } (h'', \mu ', \texttt {c}', \alpha ) \quad \chi \xrightarrow {\sigma } \chi '\end{array}}{\begin{array}{c}(\chi , h', \mu , \texttt {c}, \alpha ) \xrightarrow {\sigma } (\chi ', h'', \mu ', \texttt {c}', \alpha )\end{array}} $$

$$ \frac{\begin{array}{c}(h', \mu , \texttt {c}, \alpha ) \rightarrow (h'', \mu ', \texttt {c}', \alpha ')\end{array}}{\begin{array}{c}(\chi , h', \mu , \texttt {c}, \alpha ) \rightarrow (\chi , h'', \mu ', \texttt {c}', \alpha ')\end{array}} $$

#### Example:

Let us revisit the Treiber Stack and in particular the violating execution described in Fig. 2. The violating history consists of two sessions, with one session containing the invocation push(1) and another containing pop. The execution of push(1), following the language semantics, creates the events $\sigma _1$ and $\sigma _2$ such that $act(\sigma _1) = \mathsf {W}(L.\texttt {Val},1)$ and $act(\sigma _2) = \mathsf {U}(\texttt {Top}, \texttt {NULL}, \mathsf {L})$ which are both added to the store state. The execution of pop generates the read event to Top, which following the store semantics picks the set $\varSigma ' = \{\sigma _2\}$, resulting in read event $\sigma _3$ such that $act(\sigma _3) = \mathsf {R}(\texttt {Top}, \mathsf {L})$. Under EC, the following read to L.Val by pop is unconstrained and hence simply picks $\varSigma ' = \phi$, resulting in the event $\sigma _4$ such that $act(\sigma _4) = \mathsf {R}(L.\texttt {Val}, 0)$ where 0 is the initial value. This results in violation of the AddRem specification.

Notice that $\mathsf {so}(\sigma _1, \sigma _2)$ and $\mathsf {vis}(\sigma _2, \sigma _3)$. Hence, under MW+MR, while generating the read event to L.Val by pop, the store must pick $\varSigma ' = \{\sigma _1, \sigma _2\}$ to satisfy the axioms of MW+MR, so that the event must read the value 1, which prevents the violation from occurring.

### Correctness Specification

Given an abstract execution obtained after executing a history on a replicated store under some consistency policy, how do we decide if it correctly obeys the semantics of the data structure implemented by the library? Linearization would require us to demonstrate a total order on all method invocations which would be admissible by a sequential reference implementation of the data structure. However, since the consistency model of a replicated system is substantially weaker than sequential consistency, it becomes necessary to also weaken correctness requirements [34, 37]. We use the axiomatic specifications of data structure correctness as proposed by Emmi et al. [17], which are equivalent to standard linearizability, as our basis, and then weaken them systematically to adapt them to be useful in a replicated environment. Axiomatic specifications do not require a total order to be established on method invocations, do not refer back to a reference implementation, and also match the axiomatic, declarative nature of the semantics of the replicated store.

First, we define all abstract executions that can be generated given a library implementation, a history and a consistency policy. The initial state of the replicated store is assumed to be empty, i.e. $\chi _{\mathsf {Init}} = (\phi ,\, \phi ,\, \phi ,\, \phi )$. Let $h_{\epsilon }$ be the empty history which associates an empty sequence ($\epsilon$) of invocations to each session. Let $\texttt {c}_{\mathsf {Init}}$ be the initial implementation state which simply associates the empty program $\epsilon$ to each session.

#### Definition 1

Given a set of sessions *S*, a history *h*, a library implementation *L* and a consistency policy $\varPsi$, the abstract executions generated by $\varOmega _{h, L, \varPsi }$ are defined as : $\llbracket \varOmega _{h,L,\varPsi } \rrbracket \! = \{\varGamma \, |\, (\chi _{\mathsf {Init}}, h, (\phi , \phi ), \texttt {c}_{\mathsf {Init}}) \rightarrow ^{*} (\_, h_{\epsilon }, \varGamma , \_)\}$

Thus, executing all invocations in the history under a given consistency policy and library implementation gives rise to the set of final abstract executions. Due to the non-deterministic nature of the semantics, multiple abstract executions could be generated. Correctness of an abstract execution is specified in terms of various **axioms** that it must obey. An implementation is correct under a consistency policy if for all possible histories, all final abstract executions generated by the implementation obey the axioms.

To illustrate, let us consider the Stack data structure. It has two methods $M=\{\texttt {Push}, \texttt {Pop}\}$. Given a method invocation event $\gamma = (i,m,a,r,s)$, we assume projection functions for all the respective components (e.g., m, a, and r). Further, we assume a $\mathsf {match}$ predicate relating two method invocation events defined thus:

$$\begin{aligned} \mathsf {match}(\gamma _1, \gamma _2) \Leftrightarrow \mathsf {m}(\gamma _1) = \texttt {Push} \wedge \mathsf {m}(\gamma _2) = \texttt {Pop} \wedge \mathsf {a}(\gamma _1) = \mathsf {r}(\gamma _2) \end{aligned}$$

Let $\texttt {EMPTY}$ denote a special value signifying the empty return value (see, e.g. the Treiber Stack impl. in Fig. 1). Consider an abstract execution $\alpha = (\varGamma , \mathsf {so}_{\varGamma })$. We define the happens-before relation for method invocations as $\mathsf {hb}_{\varGamma } = (\mathsf {match} \cup \mathsf {so}_{\varGamma })^{+}$. Then, the correctness of $\alpha$ can be specified in terms of the following axioms:

- $$\mathsf {AddRem}$$ : $$\forall \gamma \in \varGamma . \mathsf {m}(\gamma ) = \texttt {Pop} \wedge \mathsf {r}(\gamma ) \ne \texttt {EMPTY} \Rightarrow \exists \gamma ' \in \varGamma . \mathsf {match}(\gamma ', \gamma )$$
- $$\mathsf {Injective}$$ : $$\forall \gamma _1, \gamma _2, \gamma _3 \in \varGamma . \mathsf {match}(\gamma _1, \gamma _2) \wedge \mathsf {match}(\gamma _1, \gamma _3) \Rightarrow \gamma _2 = \gamma _3$$
- $$\mathsf {Empty}$$ : $$\forall \gamma _1, \gamma _2, \gamma _3 \in \varGamma . \mathsf {m}(\gamma _1) = \texttt {Pop} \wedge \mathsf {r}(\gamma _1) = \texttt {EMPTY} \wedge \mathsf {m}(\gamma _2) = \texttt {Push} \wedge \mathsf {hb}_{\varGamma }(\gamma _2, \gamma _1) \Rightarrow \exists \gamma _3 \in \varGamma . \mathsf {match}(\gamma _2, \gamma _3)$$
- $$\mathsf {LIFO-1}$$ : $$\forall \gamma _1, \gamma _2, \gamma _3 \in \varGamma . \mathsf {m}(\gamma _1) = \texttt {Push} \wedge \mathsf {match}(\gamma _2, \gamma _3) \wedge \mathsf {hb}(\gamma _2, \gamma _1) \wedge \mathsf {hb}(\gamma _1, \gamma _3) \Rightarrow \exists \gamma _4 \in \varGamma . \mathsf {match}(\gamma _1, \gamma _4)$$
- $$\mathsf {LIFO-2}$$ : $$\forall \gamma _1, \gamma _2, \gamma _3, \gamma _4 \in \varGamma . \lnot (\mathsf {match}(\gamma _1, \gamma _4) \wedge \mathsf {match}(\gamma _2, \gamma _3) \wedge \mathsf {hb}(\gamma _2, \gamma _1) \wedge \mathsf {hb}(\gamma _3, \gamma _4) \wedge \mathsf {hb}(\gamma _1, \gamma _3))$$

These axioms follow from those given in [17], except that instead of using a linearization order as done in [17], we use a weaker happens-before $\mathsf {hb}_{\varGamma }$ order. It is also possible to use the even weaker session order $\mathsf {so}_{\varGamma }$ in place of $\mathsf {hb}_{\varGamma }$. We have already seen the $\mathsf {AddRem}$ axiom in §2. The $\mathsf {Injective}$ axiom enforces that an element pushed onto the stack is not popped more than once^4^. The $\mathsf {Empty}$ axiom says that if a pop invocation ($\gamma _1$) returns EMPTY and if there is a push invocation ($\gamma _2$) that happens-before it, then $\gamma _2$ must be matched to another pop. This reflects the expected stack-like behavior from the point of view of a client who observes these invocations. The $\mathsf {LIFO-1}$ property specifies that if a push invocation $\gamma _2$ happens-before another push invocation $\gamma _1$, with both of them happening-before a pop invocation $\gamma _3$, and if $\gamma _2$ is matched with $\gamma _3$, then to respect the LIFO order, $\gamma _1$ must also be matched (to some $\gamma _4$). $\mathsf {LIFO-2}$ complements $\mathsf {LIFO-1}$ by requiring that $\gamma _3$ cannot happen-before such a $\gamma _4$. The specifications for other data structures we have considered, including Queue and Exchanger can be found in [32].

## Bounded Verification

We now present an automated bounded verification procedure capable of generating abstract executions that violate data structure correctness specifications under a given consistency policy. We take advantage of the axiomatic nature of both the semantics and specification and reduce the problem to that of checking the satisfiability of a collection of formulae in first-order logic (FOL), which can be dispatched to an off-the-shelf SMT solver. In particular, our strategy is to instantiate a bounded number of invocations (*k*) without specifying their method types, arguments, or session information, and instead leave it upto the solver to search efficiently among all histories of length *k*.

### Vocabulary

Given a library $L = (\mathsf {M}, \mathsf {Impl})$, we first take each method implementation and unroll loops upto a constant bound^5^, and give a label to each program statement that interacts with a replicated location (e.g. see the Treiber Stack impl. in Fig. 1). Let $\mathbb {L}$ denote this set of labels.

We use an uninterpreted, finite sort $\mathsf {I}$ to represent invocations in the history that we wish to construct, and then constrain this sort to contain only the distinct elements $\mathsf {INV}_1, \ldots , \mathsf {INV}_k$. In addition, we use uninterpreted sorts $\mathsf {E}$ and $\mathsf {V}$ to represent the set of replicated store events and values that are read or written by them. We define the function $\mathsf {meth} : \mathsf {I}\rightarrow \mathsf {M}$ to associate a method type with each invocation. We use an uninterpreted sort $\mathsf {S}$ to denote the set of sessions involved in the history. The function $\mathsf {sess} : \mathsf {I}\rightarrow \mathsf {S}$ associates a session with each invocation.

For each method $m \in \mathsf {M}$ and each program statement labeled *n* in the implementation $\mathsf {Impl}(m)$, we define the function $\mathsf {P}_{mn}:\mathsf {I}\rightarrow \mathsf {E}$ to associates the event generated by the program statement to an invocation. In addition, functions $\mathsf {arg},\mathsf {ret} : \mathsf {I}\rightarrow \mathsf {V}$ associate the argument and return values to each invocation. For every local variable v used in a program, function $\rho _{\texttt {v}} : \mathsf {I}\rightarrow \mathsf {V}$ denotes the value of the local variable in that invocation. The predicate $\mathsf {so}_{\mathsf {I}} : \mathsf {I}\times \mathsf {I}\rightarrow \mathbb {B}$ denotes the session order relation among invocation instances.

We define functions $\mathsf {loc},\mathsf {rval},\mathsf {wval} : \mathsf {E}\rightarrow \mathsf {V}$ to associate locations, values read and values written by events resp. We use the uninterpreted, finite sort $\mathbb {E}$ containing elements $\mathsf {R}, \mathsf {W}, \mathsf {U}$ to denote various event types. The function $\mathsf {Etype} : \mathsf {E}\rightarrow \mathbb {E}$ associates the type with each event. Finally, predicates $\mathsf {vis}, \mathsf {ar}, \mathsf {so}_{\mathsf {E}}, \mathsf {rf} : \mathsf {E}\times \mathsf {E}\rightarrow \mathbb {B}$ denote the visibility, arbitration, session order, and read-from relations resp. among events.

For every replicated location, we also instantiate a distinct value referring to the location. For example, for the Treiber Stack implementation (Fig. 1), we have distinct values for Top and for the Val and Next fields of each New Node generated by an invocation. Since the number of invocations is fixed (*k*), the number of such locations to be instantiated can also be pre-determined statically. We also define a function $\mathsf {Initval} : \mathsf {V}\rightarrow \mathsf {V}$ which fixes an initial value for every location, and assigns initial values to all locations used in the execution.

### Implementation Constraints

We now describe constraints on the events imposed by the implementation. First, note that even though the set of functions $\{\mathsf {P}_{mn} | m \in \mathbb {M},\ n \in \mathbb {L}\}$ are defined for every invocation, an invocation $\mathsf {i}$ will only have a fixed method type $\mathsf {meth}(\mathsf {i})$, and hence will only generate events corresponding to program statements in the implementation of $\mathsf {meth}(\mathsf {i})$. We designate a special event $\bot : \mathsf {E}$ and associate it for program statements of every other method type using the following constraint:

$$ \forall i \in \mathsf {I}\ \forall m \in \mathbb {M}\ \forall n \in \mathbb {L}.\ m \ne \mathsf {meth}(i) \Rightarrow \mathsf {P}_{mn}(i) = \bot $$

For program statements in the implementation of $\mathsf {meth}(\mathsf {i})$, we add constraints for every statement based on its type. Note that loops have already been unrolled and for every statement labeled *n* in method $\mathsf {m}$, we collect the conditionals of any if statement enclosing the statement and replace any local variable $\texttt {v}$ used in those conditionals with the corresponding function $\rho _{\texttt {v}}(\mathsf {i})$ (for invocation $\mathsf {i}$) to obtain the formulae $\llbracket \phi _{mn} \rrbracket \!_{\mathsf {i}}$. To illustrate the constraints added for different types of statements, consider the rule for reads:

$$ \frac{\begin{array}{c}\mathsf {Impl}(m):n:\ \texttt {v} = \texttt {l}\end{array}}{\begin{array}{c}\forall i \in \mathsf {I}.\ (\mathsf {meth}(i) = m \wedge \!\llbracket \phi _{mn} \rrbracket \!_{i}) \Rightarrow (\mathsf {Etype}(\mathsf {P}_{mn}(i)) = \mathsf {R} \wedge \mathsf {loc}(\mathsf {P}_{mn}(i)) = \texttt {l} \\ \wedge \mathsf {rval}(\mathsf {P}_{mn}(i)) = \rho _{\texttt {v}}(i)\end{array}}) $$

The rule essentially specifies the constraint for statement labeled $\mathsf {n}$ in the implementation of method $\mathsf {m}$ if it is a read operation. The constraint appropriately sets the $\mathsf {Etype}$, $\mathsf {loc}$ and $\mathsf {rval}$ functions of event $P_{mn}(i)$ for every invocation *i*, if the invocation has a method type of $\mathsf {m}$ and the enclosing if conditionals (if any) are satisfied. The rules for write and CAS statements are similar (they also set the $\mathsf {wval}$ function and additionally CAS also checks whether the value read is equal to its first argument) and can be found in [32]. In addition, we also relate adjacent events of the same invocation with the session order relation $\mathsf {so}_{\mathsf {E}}$.

### Abstract Execution Constraints

On encountering a return statement, we record the returned value using the following constraint:

$$ \frac{\begin{array}{c}\mathsf {Impl}(m):n:\ \texttt {return } \texttt {v}\end{array}}{\begin{array}{c}\forall i \in \mathsf {I}.\ (\mathsf {meth}(i) = m \wedge \llbracket \phi _{mn} \rrbracket _{i}) \Rightarrow (\mathsf {ret}(i) = \rho _{\texttt {v}}(i) \wedge \mathsf {completed}(i))\end{array}} $$

Apart from setting the $\mathsf {ret}$ value, we also use another unary predicate $\mathsf {completed}$ to encode that the invocation has completed and reached the return statement. This is needed because we are unrolling loops upto a fixed bound. Since we know the last program statement statically, if we encounter this statement without reaching return for an invocation, then $\mathsf {completed}$ will be set to $\mathsf {false}$.

We also encode the constraint that the session order relation ($\mathsf {so}_{\mathsf {I}}$) among invocations of the same session is a total order. Finally, we also encode that if two invocations $\mathsf {i}_1$ and $\mathsf {i}_2$ are in session order ($\mathsf {so}_{\mathsf {I}}(\mathsf {i}_1, \mathsf {i}_2)$), then the last event of $\mathsf {i}_1$ and the first event of $\mathsf {i}_2$ are in event session order ($\mathsf {so}_{\mathsf {E}}$).

### Replicated Store Constraints

We must also encode constraints ensuring that the semantics of the replicated store are preserved. First, we capture various properties of relations on events, viz. $\mathsf {vis}$ is anti-symmetric and irreflexive, $\mathsf {ar}$ among write events to the same location is a total order, $\mathsf {vis}$ and $\mathsf {so}_{\mathsf {I}}$ do not clash with each other, $\mathsf {ar}$ does not clash with $\mathsf {vis}$ and $\mathsf {so}_{\mathsf {I}}$. All these constraints are implicitly enforced by the semantics of the replicated store, so that the state of the store reached after any number of execution steps must obey them.

The various consistency policies in Table 1 can be directly encoded using the relations defined in the vocabulary. We now turn to encoding the last-writer-wins nature of the data store, which relates the $\mathsf {vis}$ and $\mathsf {ar}$ relations with the read and write values ($\mathsf {rval}$ and $\mathsf {wval}$) of the events.

$$\begin{aligned} \begin{array}{lcl} \forall e_1,e_2 \in \mathsf {E}. \mathsf {rf}(e_1, e_2) &{} \Rightarrow &{} \mathsf {vis}(e_1, e_2) \wedge \mathsf {wval}(e_1) = \mathsf {rval}(e_2) \wedge \\ &{} &{} \forall e_3 \in \mathsf {E}_{\mathsf {W}}^{\mathsf {loc}(e_2)}. (\mathsf {vis}(e_3, e_2) \Rightarrow e_3 = e_1 \vee \mathsf {ar}(e_3,e_1)) \end{array} \end{aligned}$$

$$ \forall e_1 \in \mathsf {E}_{\mathsf {R}}. (\forall e_2 \in \mathsf {E}. \lnot \mathsf {rf}(e_2, e_1)) \Rightarrow \mathsf {rval}(e_1) = \mathsf {Initval}(\mathsf {loc}(e_1)) $$

In the above constraints, we use the notation $\mathsf {E}_{\mathsf {W}}^{\mathsf {l}}$ to indicate only those events that write to location $\mathsf {l}$, and $\mathsf {E}_{\mathsf {R}}$ for read events. The first constraint enforces the reads-from event to be the most recent visible event according to the arbitration order, and also constrains the read value. The second constraint disallows out-of-thin-air reads by enforcing that if there are no $\mathsf {rf}$ events, then the value read must be the initial value. As an optimization, while encoding this constraint in our tool, we enumerate all possible write events to the same location (which are guaranteed to be finite since we only have *k* invocations) in the antecedent, instead of the universal quantification used above.

For CAS operations which generate update events, we encode the constraint (as derived from the semantics rule R-CAS) that two update events should not read from the same event:

$$\forall e,e_1,e_2 \in \mathsf {E}.\ \mathsf {Etype}(e_1) = \mathsf {U} \wedge \mathsf {Etype}(e_2) = \mathsf {U} \wedge \mathsf {rf}(e,e_1) \wedge \mathsf {rf}(e,e_2) \Rightarrow e_1 = e_2 $$

### Specification Constraints

The axioms of correctness for data structures only use an invocation’s argument and return values, and the session order relation among invocations in the abstract execution. Thus, they can be directly encoded using our vocabulary. Given an axion $\theta$, we encode its negation to find histories which have abstract executions that violate the axiom.

For example, to find violations of the $\mathsf {AddRem}$ axiom, we add the following constraint:

$$ \exists \mathsf {i}_1 \in \mathsf {I}.\ \mathsf {meth}(\mathsf {i}_1) = \texttt {POP} \wedge \mathsf {ret}(\mathsf {i}_1) \ne \texttt {EMPTY} \wedge \forall \mathsf {i}_2 \in \mathsf {I}.\ \lnot \mathsf {match}(\mathsf {i}_2, \mathsf {i}_1) $$

where we use the predicate $\mathsf {match}: \mathsf {I}\times \mathsf {I}\rightarrow \mathbb {B}$ defined in a similar manner as in Sect. 3.4. This completes the entire description of our encoding.

Our main soundness result can be formalized thus^6^

#### Theorem 1

Given a library implementation *L*, consistency policy $\varPsi$ and a correctness axiom $\theta$, if the collection of formulae described above are satisfiable, then there exists a history *h* and an abstract execution $\varGamma \in \llbracket \varOmega _{h,L,\varPsi } \rrbracket$ which violates $\theta$.

## Experimental Evaluation

**Table 2.** Consistency policies required for various implementations and specifications.

Benchmark	AddRem	Injective	Empty[SO]	Empty[HB]	FIFO-1/LIFO-1/Exchange	FIFO-2/LIFO-2	Max time (s)
2Lock Queue [29]	MW+MR	MW+MR +WFR	CC	CC	MW+MR	MW+MR	269
LockFree Queue [29]	MW+MR	EC	CC	CC	MW+MR	EC	152
HW Queue [22]	EC	EC	RMW	MW+MR +RMW	CC	MW+MR	61
Treiber Stack [40]	MW+MR +WFR	EC	CC	CC	MW+MR +WFR	EC	245
Elimination Stack [20]	MW+MR +WFR	EC	CC	CC	MW+MR +WFR	MW	65
Exchanger [20]	MW	EC	-NA-	-NA-	MW	-NA-	40

We have implemented our bounded verification procedure and applied it to a number of library implementations that have been widely-used in the world of shared-memory systems. We generate FOL formulae for each implementation as described in Sect. 4 and dispatch them to Z3 to determine their satisfiability. For queues, we have used the 2LockQueue, LockFree Queue and Herlihy and Wing (HW) Queue implementations, while for stacks, we have applied our approach on the Treiber and Elimination Stack implementations. The Elimination stack uses the exchanger implementation, and so we have also checked the correctness of the exchanger.

Since our analysis takes as input the bound on the number of invocations (*k*), the consistency policy, and the specification, we deploy the system as follows: For each implementation and specification pairing, we start with bound $k = 2$ and the weakest consistency policy (EC). If we do not find any violation, then we increase the bound by 1 and perform the analysis again. On the other hand, if we do find a violation, then by Theorem-1, we know that it is guaranteed to be an actual violation. We record its structure from the satisfiable model returned by Z3, and then increase the consistency policy to the next higher level. We continue this process until we exhaust our verification time budget (of 1 hour per benchmark implementation). Note that all the consistency policies that we consider can be arranged in a lattice [38] whereby the higher one goes up the lattice, the consistency policies become stronger, which means they allow only a subset of executions that are allowed by policies weaker than them. Our tool automatically traverses this lattice to find the weakest consistency policy at which no bounded violation is found.

Table 2 summarizes the results of this process. For each pair of benchmark implementation and correctness specification, it shows the weakest consistency policy at which we did not find any violations. This means that at every consistency policy weaker than the one specified in the table, violations were discovered. For each benchmark, we also note the maximum time needed to find a violation for any specification by Z3. Some specifications were discussed in §3.4, with Empty[SO] meaning we replace the relation $\mathsf {hb}_{\varGamma }$ with $\mathsf {so}_{\varGamma }$ in the specification; the correctness specifications for Queues and Exchangers are given in [32]. Across all benchmarks, we found that the longest history which violated any specification within the time bound considered consisted of 6 invocations.

To empirically validate our results, we also executed all the benchmarks at the appropriate consistency levels on Cassandra, a real-world replicated data store. We configured Cassandra with 3 replicas running on Amazon EC2 instances at different physical locations (all on the US East Coast). We randomly generated client invocations at all 3 replicas and ran each implementation for 4 h (on average 92000 invocations/benchmark). We collected the resulting traces and checked the specifications. We did not find any violation of the specifications, and surmise that violations, when they do occur, manifest in smaller executions that can be systematically checked by our analysis.

The results yield a number of interesting observations. First and foremost, note that even for the same benchmark, different correctness specifications require different consistency policies, ranging from the weakest, *Eventual Consistency*, (EC) to the strongest, *Causal Consistency*, (CC). This suggests that depending upon the requirements of the clients of the library, there is a trade-off between consistency and correctness that can be effectively explored. It has long been known that *Causal Consistency* incurs a performance penalty [3] due to expensive dependency tracking, significant metadata storage, and long wait times for all causally dependent data to arrive. A number of recent approaches [9, 14, 28] have looked at improving the performance of *Causal Consistency*, mainly by reducing the amount of dependent data required. Our experiments suggest that many important correctness properties of library implementations may not require CC, but would work correctly under weaker session guarantees or even EC. Note that as we discussed in Sect. 2, MW+MR only require all data to be propagated from the same session, while MW+MR+WFR requires data to be propagated across the entire causal chain.

Another interesting observation is that important properties such as Injective and FIFO/LIFO only require EC for most benchmarks. We also notice that for the same correctness specification, different benchmarks require different consistency policies, especially among the various Queue benchmarks. This illustrates that clients have flexibility in choosing an implementation, based on the properties that they need. For example, an HW queue can satisfy the AddRem specification at the weakest consistency policy (EC), but requires CC for FIFO-1, which can be satisfied using just session guarantees by both 2LockQueue and LockFreeQueue. No single queue implementation provides all correctness guarantees at the weakest consistency level. For stacks, the Elimination Stack and the Treiber Stack require the same consistency policies for every specification except LIFO-2, for which the Elimination Stack requires MW for the Exchange property of the underlying Exchanger to be satisfied. By analyzing violations, we also found that both the access pattern of different implementations as well as the semantics of the data structure (stack vs. queue) played a major role in determining how and if violations occur.

Note that even though we unroll loops upto a fixed bound, for all benchmarks except LockFree Queue, the unrolling factor does not matter because in every loop, every iteration except the last only performs read events, and the values read are only used in the same iteration. Hence, only the last iteration which performs a write/update event is relevant; unrolling the loop once is sufficient.

**Fig. 4.**
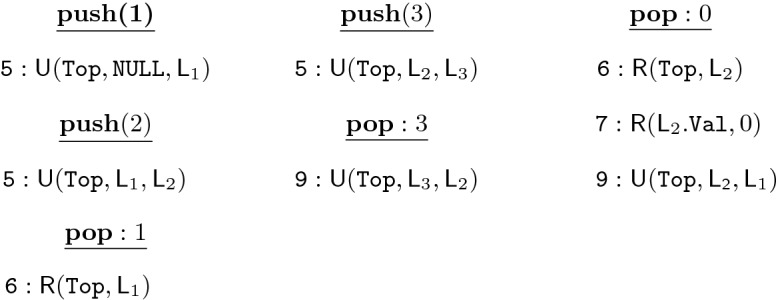
A violation of $\mathsf {LIFO-1}$ by Treiber Stack under MW+MR involving 6 invocations

In order to illustrate the complex violations automatically generated by our framework, consider the violation of LIFO-1 in the Treiber stack implementation under MW+MR in Fig. 4. Here, invocations in the same column are in the same session. Following the notation as used in the specification in Sect. 3.4, $\gamma _1 = \texttt {push}(2)$, $\gamma _2 = \texttt {push}(1)$, $\gamma _3 = \texttt {pop}:1$. As a concrete violation of the specification, $\gamma _2$ happens before $\gamma _1$, but $\gamma _3$ returns the value pushed by $\gamma _2$ even though $\gamma _1$ is unmatched, thus disobeying the LIFO property. The reason behind this violation is that another pop operation (pop:0) is actually popping the element pushed by push(2), but it does not read the value 1 and instead reads the initial value 0 (thus also violating AddRem). As a result, the last pop operation in the leftmost session sees only the element 1 on the stack. We note that there is no violation of smaller length under MW+MR. By upgrading the consistency level to MW+MW+WFR, the violation is eliminated.

## Related Work and Conclusion

Verifying applications under weak consistency has received significant attention in recent years. A number of efforts [2, 19, 23, 25, 38] have looked at the problem of verifying arbitrary safety invariants while others have considered verification with respect to distributed database applications and specific high-level transactional properties [5–7, 10, 30, 35]. These results are orthogonal to the work described here, since neither consider the question of safely migrating performant concurrent libraries to a replicated environment.

More directly related are proposals to deal with the specification and verification of various properties of CRDTs [12, 18, 31, 41, 42]. CRDTs also offer a library interface to clients and have been implemented for various data structures such as set, list, map, etc. They follow a different system model than the library implementations that we have considered in our work, and typically do not require any form of synchronization. However, this requirement imposes stringent constraints on their design (for example, in an op-based CRDT, all operations have to commute with each other). We are not aware of any CRDT-like implementation of concurrent data structures such as Queue, Stack and Exchangers that we have considered here.

Prior works [18, 31] have also developed automated or semi-automated approaches to verify the convergence of CRDTs, an important but fairly low-level property that does not shed much insight on the correctness of libraries built using them. High-level correctness specifications of CRDTs are either given in terms of abstract RDT specifications [12, 42] or customized specification frameworks such as replication-aware linearizability [41]. Both of these specification styles are closer to linearizability, but since direct linearization of all operations an execution is not possible in a distributed environment, both approaches allow relaxations to help decide a linearization order. These relaxations typically take the form of allowing different per-invocation linearizations based on the type of the invocation and the visibility relation. This can lead to complicated specifications that can be substantially different from their shared-memory counterparts, complicating verification. In contrast, our axiomatic style also allows clients of the library to know exactly how the relaxations in a replicated environment will impact observable behavior. Finally, unlike other prior work, we develop a fully automated approach for bounded verification of library implementations.

There has also been recent interest in specifying and verifying concurrent library implementations for shared memory systems [16] and weak memory models [15, 34]. While the specification style of weak memory models bears some superficial resemblance to that of weak consistency, the underlying system model is quite different, and weak consistency models allows relaxed behaviors which are not allowed by weak memory models. They also offer more fine-grained control than possible under weak memory given their ability to provide session-level as well as system-wide consistency guarantees to individual low-level operations. [34] proposes axiomatic specifications of libraries using happens-before and program orders. Our specifications, while similar in spirit, are more fine-grained and better suited to replicated systems.

To conclude, we tackle the problem of migrating concurrent library implementations from shared-memory systems to replicated, distributed ones. We define a sensible semantics for such implementations on a replicated store parametric in the consistency policy of the store and describe how to migrate the correctness specifications for such libraries with minimal changes. Our verification framework automatically finds bounded violations of these specifications. Parametericity of consistency policies in the analysis allows us to find the weakest policy that eliminates a discovered violation. Our experiments have demonstrated that the proposed framework is effective in finding non-trivial violations in a number of challenging and diverse benchmarks. We also find that the spectrum of weak consistency policies in replicated systems can be effectively explored to tradeoff correctness and performance.

## Footnotes

1 CAS operations are typically supported in replicated systems by providing transactional guarantees to a group of operations; e.g., lightweight transaction support provided in Cassandra [26].

2 We formalize all consistency policies used in the paper in the next section.

3 Note that the lack of any constraints (i.e. $\varPsi = true$) corresponds to Strong Eventual Consistency [18]. Since we assume SEC, our definition of Causal Consistency corresponds to Causal Convergence (CCv) as defined by [8].

4 Note that we assume all methods are called with distinct arguments.

5 Loops are typically only used to busy wait for a successful CAS operation in the applications we consider.

6 A Proof Sketch can be found in [32].
